# Surgical Operations in Private Practice

**Published:** 1904-12-17

**Authors:** John Aikman

**Affiliations:** Glas.


### Hospital Clinics.

### SURGICAL OPERATIONS IN PRIVATE PRACTICE.

By John Aikman, M.D., C.M., Glas.

### V.—Varicose Veins.

In the humdrum of general practice there are few more intrusive and irritating subjects than the treatment of varicose veins. All patients have some friends who have suffered severely, and few people are free from some veins which are unduly prominent. One patient's argument is that a friend was cured of severe varix by operation; another argues that a friend of his got well by some appliance which did not lay him up and caused no loss of time. The case upon which an opinion is asked may be suitable for one or the other treatment, but that is vague in the patient's mind which, for comprehensible reasons, may be in opposition to that of his medical adviser. To the patient a dilated vein is a varicose vein, and if he turns to his etymological dictionary he finds confirmation of his view. Every dilated vein has not undergone the changes which mark the disease varix, though it has come within the conditions which favour them. The man whose nervous energy drives him to put the fullest stress upon his venous system is the very man who is most intolerant of all but radical measures for cure. The veins of the leg, the anus, and the spermatic cord are those which are most liable to physiological distension; they are also the most frequent sites of varix.

But another factor cannot be disregarded. If the blood which they carry is impure, it has a greater opportunity to permeate the walls of the vessels and set up the irritation which results from chemical or bacterial products. It is a parallel to the frequency of gouty changes in the great toe, where the circulation is at its slowest. The veins at the anus become dilated when the portal circulation is impeded and the intestinal contents have become fœtid. The veins of the spermatic cord become dilated when the testicle is receiving an additional blood supply, and is returning venous blood charged with the products of the newly acquired function of the gland. It will be curious to note the effect of a recently suggested treatment for rheumatoid arthritis on the veins. It consists in stopping the return of the venous blood from the affected limb until œdema has taken place, and then removing the ligature, in the hope that the effused plasma will have dissolved and will carry with it some of the deposited salts. Experience is wanted on this point.

The history of pregnancy is interesting from the converse point of view. No woman is free from dilatation of the veins of her legs in the later stage of her pregnancies; by no means every woman has a resulting varix. There are great changes in the blood of a pregnant woman, but they are physiological; it would seem as if some added pathological element was needful to set up the disease varix.

Another factor of some importance is the age at which varix shows itself. Most serious cases appear early in life, before wear and tear has justified loss of tone in the affected vessels; and there is often a history of the same or some allied ailment in near relatives. It is no argument to the contrary to assert that the number of varicose persons increases with age, for the personally acquired blood impurities increase in a far greater ratio. In what might be called acquired cases, the veins about the anus are the earliest to show signs of phlebitis and thrombosis. If the vein is opened and the clot turned out, the condition shows little tendency to spread, provided that the wound is kept clean and the fault in the portal circulation corrected. Next in order of favourable prognosis is varicocele; if the recent function of the testicle is the only cause of the thickening of the veins of the spermatic cord, rest, with time to become accustomed to the altered blood, is all that is required; thrombosis is very rare.

But in the presence of gonorrhœal infection the behaviour of the dilated veins is different; they may form the starting-point of epididymitis or orchitis. Thrombi then form in them and are a fertile source of mischief.

The operation for varicocele is required by the regulations which guard entrance into some of the public services; it is easy of performance if aseptic precautions are observed. Shaving and effective cleansing are very needful, and it is a good precaution against reinfection of the wound to stitch the prepuce to the skin of the opposite thigh. For the same reason it is desirable to have the incision as high as possible, and to draw the cord and its veins up to the incision. The vas is easily recognised from its firmness to the touch, and near it lie the spermatic artery and nerve. The next point is to unravel the plexus of veins, with a view to spare such as are yet normal. The abnormal veins are then gathered into a bundle: the useless portion to be excised, the remainder to form a suspensory ligament for the testicle. The lower ligature should be near the testicle; by traction on it the proper height of the testicle may be determined and the site of the upper ligature. The lower ligature is then cut short, and the free end of the upper passed under it and through the mass of veins below it, care being taken that it has sufficient hold not to slip under the strain of the weight of the testicle. The condemned portion of the veins is then excised, leaving a stump long enough to insure against slipping. The free ends of the upper ligature can now be made to approximate the stumps of the bundle of veins, and tied with no more firmness than will secure co-aptation. A suspensory bandage is needful for six weeks or two months, when all artificial support may be discontinued. I have always used silk ligatures, and I think that they last longer than catgut, and are harmless if the silk thread is made to bear upon the silk binding of the lower ligature.

Dilated veins in the legs present some new features. There is always some thickening of their coats, in simple cases chiefly of the middle one. There is always lengthening, and consequent tortuosity; the smaller veins appear like pencil-marks on the surface of the skin, not invariably to its damage. Thrombosis and attachment of the larger veins to the skin are more certain signs of true varix. There seems to be a thickening of the middle coat and some degeneration of the inner coat, which makes it liable to fracture at the points of flexure of the limb or at the tortuosities of the vein, and where such fracture takes place we have thrombosis within the vein, and adhesion around it. This varicose process is apt to appear in patches at first; its tendency is to spread upwards rather than downwards, and the cause of its extension would seem to be dilatation and the character of the contained blood. Gout is certainly a frequent cause, and I think that all changes in the kidney which affect the general health predispose to it. There is little tendency to varix in the veins which lie under the tougher fasciæ of the limbs, and in which the blood is pressed rapidly onwards by the swelling of the muscles during their action. A similar condition is produced when we apply a crêpe bandage to the limb: the muscles press the veins against it, and if the valves are competent no stagnation is possible. Elastic stockings are not so good; the pressure is apt to be too great, and, after wear, tends to become unequal.

The earlier surgical measures for the treatment of varicose veins dealt chiefly with the diseased area, and were often disappointing. More than 20 years since Lord Lister mentioned to me the surprising results which he had obtained as following the excision of portions of the saphena vein in the thigh in cases which were too widely spread to justify large excisions in the diseased area; and he added that we still had something to learn of the proper site for operation. In the *Lancet* of April 8, 1899, and, later, in the *Lancet* of March 1, 1902, Mr. Pearce Gould brought Trendelenburg's operation under the notice of British surgeons. This operation, for details of which see the articles mentioned, is a great advance upon all former procedures; the incisions are made far outside the diseased area, the healing is more safe and rapid, the diseased veins are put at rest, and the after-results are more wide and lasting. In some cases, in which the varix did not extend very far up in the thigh, I have tied the vein a little lower down. At the apex of Scarpa's triangle the sartorius muscle crosses the adductor longus; with the upper point of contact as centre and the edge of the sartorius as guide, the vein is readily exposed and a few inches excised. If the cuts are clean and sharp the wound heals at once, and the results are better than in any operation of which I have experience for varix of the lower leg. The thickened veins lessen in the course of a few months, and in time become negligible quantities; the tendency to thrombosis disappears, and the skin, which was often on the verge of ulceration, regains its vitality. There is no objection to the excision of portions of veins or skin which may seem hopeless at the time of operation, but I advise delay in condemning even what appears very bad. A crêpe velpeau bandage should be worn for three months after operation, as in the treatment of simple dilatation, but when the skin has regained its elasticity no support is required.

